# Lavender Oil Reduces Depressive Mood in Healthy Individuals and Enhances the Activity of Single Oxytocin Neurons of the Hypothalamus Isolated from Mice: A Preliminary Study

**DOI:** 10.1155/2020/5418586

**Published:** 2020-07-14

**Authors:** Keizaburo Ogata, Koji Ataka, Hajime Suzuki, Takakazu Yagi, Ayumi Okawa, Takamasa Fukumoto, Boyang Zhang, Masanori Nakata, Toshihiko Yada, Akihiro Asakawa

**Affiliations:** ^1^Division of Clinical Psychology, Kitasato University Hospital, 1-15-1, Kitasato, Minami, Sagamihara, Sagamihara 252-0328, Japan; ^2^Department of Psychosomatic Internal Medicine, Kagoshima University Graduate School of Medical and Dental Sciences, 8-35-1 Sakuragaoka, Kagoshima 890-8520, Japan; ^3^Department of Pharmacological Sciences of Herbal Medicine, Kagoshima University Graduate School of Medical and Dental Sciences, 8-35-1 Sakuragaoka, Kagoshima 890-8520, Japan; ^4^Department of Oral and Maxillofacial Surgery, Kagoshima University Graduate School of Medical and Dental Sciences, 8-35-1 Sakuragaoka, Kagoshima 890-8520, Japan; ^5^Department of Orthodontics and Dentofacial Orthopedics, Kagoshima University Graduate School of Medical and Dental Sciences, 8-35-1 Sakuragaoka, Kagoshima 890-8520, Japan; ^6^Department of Psychology, Faculty of Law, Economics and Humanities, Kagoshima University, 1-21-30, Korimoto, Kagoshima 890-0065, Japan; ^7^Department of Physiology, Division of Integrative Physiology, Faculty of Medicine, Jichi Medical University, 3311-1, Yakushiji, Shimotsuke, Tochigi 329-0498, Japan; ^8^Department of Physiology, Wakayama Medical University School of Medicine, 811-1, Kimiidera, Wakayama 641-8509, Japan; ^9^Kansai Electric Power Medical Research Institute, 1-5-6, Minatojima-Minamimachi, Kobe 650-0047, Japan; ^10^Department of System Neuroscience, Kobe University Graduate School of Medicine, 7-5-1, Kusunoki, Chuo, Kobe 650-0017, Japan

## Abstract

**Background:**

The aim of the present study was to assess the effects of lavender oil inhalation on blood pressure, pulse measurements, cortisol levels, depressive mood, and anxiety in healthy male adults. The mechanism was investigated by the action on oxytocin single neurons in the hypothalamus of rodents.

**Methods:**

The participants (*n* = 7) were aged 20–40 years. After randomisation, they received an inhaled dose of lavender oil or distilled water for 20 min. They received the other treatment after a washout period of one week. We assessed the outcomes using the Self-Rating Depression Scale, State-Trait Anxiety Inventory, and self-rated unidimensional Visual Analogue Scale for depression; anxiety; and hunger, thirst, and appetite, respectively. Blood pressure, pulse rate, and cortisol concentration in the peripheral blood were assessed before and after inhalation. In the rodent study (*n* = 4), oxytocin single neurons were isolated from the mouse hypothalamus. Intracellular Ca^2+^ concentration in the oxytocin neurons isolated from the hypothalamus was measured following direct administration of lavender oil.

**Results:**

Seven participants completed the study. Lavender inhalation decreased Self-Rating Depression Scale score and systolic and diastolic blood pressure. *Ex vivo* administration of lavender oil increased intracellular Ca^2+^ concentration in the hypothalamic oxytocin neurons.

**Conclusions:**

Lavender oil might be a useful therapy for stress relief, and its mechanism of action may include activation of the central oxytocin neurons.

## 1. Introduction

*Lavandula angustifolia* (lavender) essential oil is approved as a herbal medicine by the European Medicine Agency and has been used as a therapeutic and cosmetic agent for centuries. Lavender oil reportedly has sedative, relaxing, and anti-infectious effects and has been shown to improve sleep quality [1, 2]. Inhalation of lavender oil has lately attracted considerable attention in aromatherapy, which is a method for reducing stress without medication. Lavender aromatherapy has been reported to decrease autonomic parameters, such as blood pressure and heart rate [3]. Lavender oil inhalation has also been reported to decrease postpartum depression [4, 5] and the anxiety levels during gynaecological examination [6]. However, several limitations are associated with these prior studies, such as the lack of a control group, and the mixing of lavender with other oils was used in the studies [4, 5]. Even where a control group was present, no vehicle was used [4–6].

Depression and anxiety disorders induced by stressful stimuli are well known to have a close relationship with the paraventricular nucleus (PVN) in the hypothalamus, which plays a pivotal role in the regulation of behaviour responses to stress via the hypothalamic-pituitary-adrenal (HPA) axis. Neuroendocrine cells synthesising corticotropin-releasing factor (CRF), oxytocin (OXT), arginine vasopressin (AVP), and brain-derived neurotrophic factor (BDNF) are located within the PVN. It has been reported that stressors activate OXT neurons in the PVN [7]. OXT neurons inhibit activation of the CRF neurons, thus leading to the suppression of HPA axis function and potentially acting as a stress antagonist [8]. Plasma OXT levels have a positive correlation with quality of life in major depressive disorder (MDD) [9] and predict the outcome of psychotherapy in chronic depression [10]. Furthermore, the effects of intranasal administration of OXT on stress-related disorders, such as anxiety, depression, and posttraumatic stress disorders, have been reported in various clinical studies [11]. However, scientific data on the mechanism of action of lavender are limited, and few studies have reported the effects of lavender on central OXT neurons.

The aims of this study were to assess the effects of lavender on autonomic parameters such as blood pressure and pulse rate, mood such as depressive mood and anxiety, and appetite scores in healthy volunteers and activity of hypothalamic OXT neurons isolated from mice.

## 2. Methods

### 2.1. Clinical Study

#### 2.1.1. Participants

Nine participants were included in the study. The inclusion criteria were as follows: subjects (1) male; (2) age: 40 ≧ aged ≧ 20 years; (3) with no serious anamnestic history (cardiovascular disease, respiratory disease, or mental health disorders); (4) healthy; (5) no experience with aromatherapy; and (6) able to participate in this experiment at the same time every other week. The exclusion criteria were as follows: subjects (1) female; (2) age: aged < 20 years or > 40 years; and (3) a serious anamnestic history (cardiovascular disease, respiratory disease, or mental health disorders). A written informed consent, indicating that the participants were able to withdraw from the study at any point if they did not agree with the study, was obtained from all the participants.

#### 2.1.2. Study Design

A preliminary study was conducted from January to February 2017. This study involved two inhalation treatments: lavender oil and distilled water (vehicle). Distilled water was chosen because it has no fragrance. A lavender-vehicle/vehicle-lavender design was adopted. Block randomisation was selected because of the small number of participants [12]. In this preliminary study, a fictitious subject was chosen via a randomisation procedure because the number of subjects enrolled in the experiment was an odd number. Assignments were randomly determined by an independent dentist in a 1 : 1 ratio in blocks. Random numbers were provided using Microsoft Excel (version 2013; Microsoft, Redmond, WA, USA). The experimental condition was written on paper in nontransparent envelopes, and the experimenter was allowed to know the experimental conditions by opening the envelope.

#### 2.1.3. Interventions

Lavender oil derived from the *Lavandula angustifolia* flower was purchased from Pranarom Co., Ltd. (Ghislenghien, Belgium). All experiments were conducted at the Kagoshima University hospital in the morning at the same time. The participants were required not to eat after 8 p.m. on the day before the examination until the end of the examination. Before starting the experiment, the participants responded to questions regarding the previous use of lavender, anamnestic history, experiment day's medical condition, and whether or not the participants were fasting. The participants underwent blood drawings, blood pressure measurements, pulse measurements, and psychological assessments. Following this, for the purpose of keeping the inhalation concentration of lavender oil constant among the participants, 75 *μ*l (1 drop) of lavender oil or distilled water was dropped from a height of less than 30 cm from the lower jaw using a micropipette (PIPETMAN P200; GILSON Inc., Middleton, WI, USA) on the dental apron placed on the participants, and they were exposed to the scent of lavender oil or distilled water through nasal or oral breathing for 20 min. After this, the participants once again underwent blood drawings, blood pressure measurements, pulse measurements, and psychological tests. Both treatment sessions were separated by a one-week washout period.

#### 2.1.4. Outcome Measurement

*(1) Psychological Questionnaires*. The Self-Rating Depression Scale (SDS), State-Trait Anxiety Inventory (STAI), and self-rated unidimensional Visual Analogue Scale (VAS) were used to assess depressive mood; anxiety; and hunger, thirst, and appetite, respectively. SDS is a widely used and an extensively researched questionnaire for the evaluation of the degree of depression [13]. In this case, we used the Japanese version of this questionnaire [14]. SDS consists of 20 items, rated on a 4-point Likert scale with total scores ranging from 20 to 80. STAI is the most commonly used self-reported state (actual, STAI-S) and trait (stable, STAI-T) anxiety scale [15]. STAI consists of 20 items each related to state and trait, rated on a 4-point Likert scale with total scores ranging from 20 to 80. In this case, we used the Japanese version of the questionnaire [16]. In this study, state anxiety was determined before and after exposure. VAS was used to evaluate the perception of hunger, thirst, and appetite. Subjects were requested to make a vertical mark on each of a 100 mm horizontal line oriented from left to right (e.g., “hungry” and “not hungry”) that best matched how they were feeling at the time. To evaluate each of the sensations, each score was determined by measuring the distance from the left side of the line to the mark.

#### 2.1.5. Cortisol Concentration in the Peripheral Blood

Blood sampling was conducted before and after exposure by a nurse, and the samples were collected using a sterilised microtube. We requested the Clinical Pathology Laboratory Co., Ltd. (Kagoshima, Japan) to measure the concentrations of cortisol (using a chemiluminescent immunoassay method).

### 2.2. Animal Experiments

#### 2.2.1. Animals

Male C57BL/6J mice at 5 to 6 weeks of age were purchased from SLC (Hamamatsu, Japan). Mice were group-housed under standard conditions at 24 ± 2°C, 50 ± 10% humidity with a 12 h/12 h light-dark cycle and ad libitum access to sterile standard chow (3.4 kcal/g; CE-2, CLEA Japan Inc., Tokyo, Japan) and water in the animal facility of the Jichi Medical University.

#### 2.2.2. Measurement of [Ca^2+^]_i_ in Single Neurons Isolated from the PVN of Mice

Measurement of the intracellular calcium ion concentration ([Ca^2+^]_i_) in single cells was carried out according to the procedure reported previously [17]. Four mice were deeply anesthetized with an intraperitoneal injection of 50 mg/kg pentobarbital. After decapitation, brain sections containing the PVN were removed from the C57BL/6 mice. The dissected brain tissues were incubated with HEPES-buffered Krebs-Ringer bicarbonate buffer solution (HKRB (mM): 129 NaCl, 5.0 NaHCO_3_, 4.7 KCl, 1.2 KH_2_PO_4_, 2.0 CaCl_2_, 1.2 MgSO_4_, and 10.0 HEPES at pH 7.4) containing 1 mM glucose and incubated in 20 U/ml papain, 0.015 mg/ml deoxyribonuclease, 0.75 mg/ml bovine serum albumin, and 1 mM cysteine in HKRB for 15 min at 36°C with shaking. The cell suspension was centrifuged at 100 ×g for 5 min. The pellet was resuspended in HKRB and distributed onto coverslips. The cells on the coverslips were kept at 20 °C in moisture-saturated dishes for 30 min. The intracellular calcium ion concentration ([Ca^2+^]_i_) in the single cells was measured using ratiometric fura-2 microfluorometry. Fura-2-acetoxymethyl ester (AM) is a membrane-permeable intracellular calcium indicator, which exhibits sensitivity to Ca^2+^ after the molecule is cleaved by an intracellular esterase. The cells were incubated with 2 *μ*M fura-2-AM for 40 min at room temperature. Fluorescence images, due to excitation at 340 and 380 nm, were detected every 8 s with an intensified charge-coupled device camera. Data were collected from cells identified as neurons by immunostaining for the neuron-specific marker microtubule-associated protein 2 and rabbit anti-OXT antibody (ab2078, abcam, 1: 1000). The F340/F380 ratio image was produced by the Aquacosmos system (Hamamatsu Photonics Co., Hamamatsu, Japan). The activity of a single cell was validated by the [Ca^2+^]_i_ response to 10^−5^ M glutamate, which was tested at the end of each measurement. A ten-thousand-fold dilution of lavender oil in 0.01% bovine serum albumin (BSA) was added in the chamber.

### 2.3. Statistical Analysis

SPSS Statistics 22 was used for statistical analysis. Differences among groups (lavender/control) and time (before/after) in SDS, STAI, VAS, blood pressure, pulse rate, and cortisol concentration in the peripheral blood were determined by two-way analysis of variance (ANOVA); multiple comparisons were tested using the Bonferroni method.

In the animal experiments, comparisons between the two groups were performed using a Chi-square analysis. Differences were considered significant at *p* < 0.05. All statistical analyses were performed using Prism 6 software (GraphPad, San Diego, CA).

### 2.4. Ethical Considerations

The clinical study was approved by the Clinical Research Ethics Committee of Kagoshima University (26–154). The animal protocols for this study were approved by the Jichi Medical University Institute of Animal Care and Use Committee. All experiments were performed in accordance with the Act on Welfare and Management of Animals (Ministry of Education, Culture, Sports, Science and Technology, Ministry of Health, Labour and Welfare, and Ministry of Agriculture, Forestry and Fisheries) and the Standards Relating to the Care and Management of Laboratory Animals and Relief of Pain (Ministry of the Environment).

## 3. Results

### 3.1. Clinical Study

#### 3.1.1. General Characteristics of the Participants

**Figure 1:**
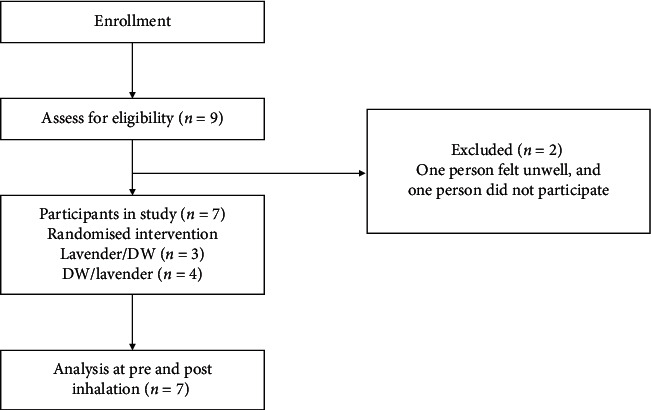
Flowchart of the randomised and crossover pilot trial involving healthy males. DW = distilled water.

Nine persons were assessed for eligibility, while two persons were excluded in this study because one felt unwell and the other decided not to participate. Seven participants out of 9 subjects met the eligibility criteria (Figure 1). One subject was unwell on the day of the experiment, and another subject was absent for personal reasons. All participants were healthy males, with a mean age of 28.0 (range 23-33-year-old). The body mass index (BMI) was 22.58 ± 0.87 kg/m^2^.

#### 3.1.2. Blood Pressure

**Figure 2:**
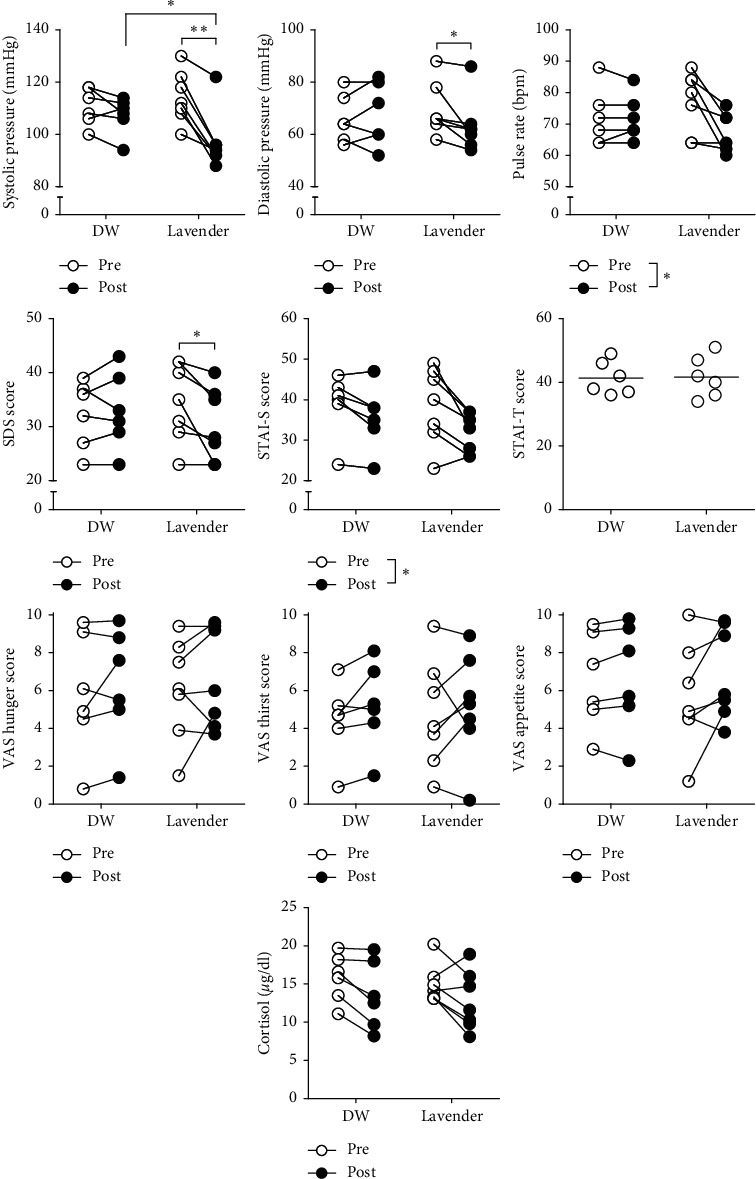
Effects of inhalation of lavender essential oils on systolic and diastolic blood pressure, pulse rate, Self-Rating Depression Scale (SDS), State-Trait Anxiety Inventory (STAI-S, STAI-T), Visual Analogue Scales (VASs; hunger, thirst, and appetite), blood cortisol levels in the peripheral blood of the participants, and distilled water (DW).

In systolic blood pressure, there was significant interaction (*F* (1, 12) = 15.41, *p* = 0.002, Table 1). In the lavender group, the postsystolic blood pressure was lower than the presystolic blood pressure and the control postsystolic blood pressure. In diastolic blood pressure, there was significant interaction (*F* (1, 12) = 5.83, *p* = 0.033, Table 1, Figure 2). In the lavender group, the postdiastolic blood pressure was lower than the prediastolic blood pressure. In pulse rate, the effect of time was observed between before and after (*F* (1, 12) = 8.52, *p* = 0.013, Table 1, Figure 2).

#### 3.1.3. Questionnaires: SDS, STAI, VAS

In SDS, there was significant interaction (*F* (1, 12) = 7.85, *p* = 0.016, Table 1, Figure 2). The post-SDS was lower than the pre-SDS. In the STAI-S, the effect of time was observed between preexposure and postexposure (*F* (1, 12) = 13.90, *p* = 0.03, Figure 2). In VAS, there was no significant difference between the lavender and the vehicle group (Table 1, Figure 2). All participants showed no changes in the STAI-T score between the 1^st^ and 2^nd^ inhalation (Table 1, Figure 2).

#### 3.1.4. Cortisol

The precortisol levels (*μ*g/dl) were 15.51 ± 1.12 in the vehicle and 14.94 ± 0.96 in the lavender inhalation group, and the postcortisol levels were 13.53 ± 1.54 in the vehicle and 12.77 ± 1.46 in the lavender inhalation group (Table 1, Figure 2). The effect of time was observed between preinhalation and postinhalation (*F* (1, 12) = 10.57, *p* = 0.007); however, there was no significant difference between the lavender and the vehicle group (Table 1, Figure 2).

### 3.2. Animal Studies

**Figure 3:**
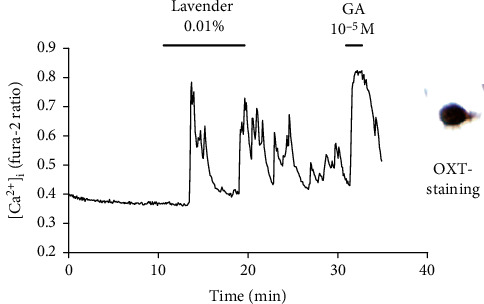
Lavender oil increases the [Ca^2+^]_i_ of oxytocin (OXT) single neurons of mouse paraventricular nucleus in the hypothalamus. GA = glutamate.

*(1) Measurement of [Ca* ^*2+*^ *]*_*i*_ *in Single Neurons Isolated from the PVN of Mice*. We isolated 150 single neurons from the PVN tissues of 4 mice and investigated whether lavender directly activated PVN neurons by measuring [Ca^2+^]_i_. Lavender oil administered at a dose of 10000-fold dilution (0.01%) increased [Ca^2+^]_i_ of OXT-positive, single PVN neurons (Figure 3). Ten out of 69 (14.5%) OXT-positive neurons and 3 out of 81 (3.7%) OXT-negative neurons were lavender-reactive, and the incidence of lavender-reactive neurons was significantly different (*χ*^2^ = 5.479, *p* = 0.0192, Table 2). The effect of lavender disappeared by addition of a perfusion solution of 0.1% BSA, which is a substance that adsorbs lavender.

## 4. Discussion

Inhalation of lavender essential oils reduced the systolic and diastolic pressures and the SDS scores in this preliminary study. The results of the present study suggest that the reduction of depressive mood and blood pressure may be achieved upon short-term exposure to lavender due to its relaxing effects. The usefulness of aromatic essential oils for inhalation in waiting rooms of mental health treatment [18] and hand massage therapy centers [19] has been reported. Aromatherapy using lavender essential oils has been applied for the treatment of various disorders and is supported by scientific evidence. The favourable effect of lavender on mood is an expected outcome of lavender intervention. Lavender has been reported to reduce depression and improve the quality of sleep and pain [1, 20]. Recently, Hassanzadeh et al. reported that aromatherapy with lavender essential oils decreased the levels of fatigue in patients undergoing haemodialysis compared to the Benson relaxation techniques [21]. Furthermore, Sánchez-Vidaña et al. indicated that inhalation of lavender essential oils ameliorates depression-like behaviour and increases neurogenesis in rats [22]. These relaxation effects of lavender are thought to arise from not only psychological effects but also physiological effects of the volatile components [23]. Lavender oil includes linalool and linalyl acetate as main components [24, 25]. Linalool from aromatic plants is under consideration as a medical ingredient. Linalool has been reported to possess various bioactivities: anti-inflammatory, anticancer, antihyperlipidaemic, antimicrobial, antinoceptive, analgesic, neuroprotective, and antidepressive properties [26]. Linalool was reported to reduce blood pressure and has been suggested to have direct effects on vascular smooth muscle leading to vasodilation in the rabbit carotid artery [27]. In addition, linalool has been demonstrated to be an antidepressant in mice [28]. Linalyl acetate has been reported to have antihypertensive properties in a hypertension-related ischaemic injury model in rodents [29] and has also been shown to induce the recovery of the acute nicotine-induced cardiovascular disruptions in rodents [30]. However, there is a lack of literature mentioning the influences of linalool, linalyl acetate, and lavender on central neurons.

Lavender oil is sensed by the olfactory epithelium, which sends signals through the limbic system to the hypothalamic tuberomammillary nucleus. This signal is then sent to the hypothalamic suprachiasmatic nucleus. Finally, this information elicits changes in autonomic nerve activity [24]. Wang et al. demonstrated that inhalation of 10% lavender activated the primary olfactory cortex, entorhinal cortex, hippocampus and parahippocampal cortex, thalamus, hypothalamus, orbitofrontal cortex, and insular cortex and its extension into the inferior lateral frontal region in healthy participants using functional magnetic resonance imaging (fMRI) [31]. These results suggest that the scent of lavender may influence the functions of central nervous system. In an animal experiment, inhalation of lavender reduced the number of c-Fos positive-cells in the PVN of the hypothalamus of mice exposed to the open field test [32]. In the present study, there was no difference in cortisol levels between the lavender and vehicle group in the clinical study. On the other hand, lavender oil directly activated OXT single neurons isolated from in the PVN of mice. PVN is the central area regulating the response to stress, and OXT neurons in the PVN regulate various stress-induced behaviours as well as CRF [33]. OXT has been reported to have antidepressant activity in animals and humans [34–36]. The mRNA expression of OXT in the PVN has been reported to decrease in rats receiving continuous restraint stress during gestation, which is a postpartum depression model. Furthermore, the administration of OXT into the PVN reversed depressive-like behaviours [37].

Intranasal transport is known to be one of the drug delivery systems to the brain which bypasses the blood-brain barrier [38, 39]. When 100 *μ*g/kg ziconotide, an analgesic agent, was administered via the intranasal route in rats (250–300 g), the concentrations in the cerebrospinal fluid (CSF) were 1.57 ± 0.44 min.µg/ml (Cmax: 33.55 ± 7.39 ng/ml) [40]. The study indicates that Cmax in CSF is 1/1000 of the dosage. In our present study, 100 nl (88.9 *μ*g)/ml of lavender oil was administered to single neurons and 75 *μ*l (66.2 mg) was inhaled by human subjects. Therefore, inhalation of lavender oil might reduce depressive mood by activating OXT neurons in the PVN.

## 5. Limitation

This study had some limitations. One of the most consistent biological findings in severe depression is the increased amount of plasma cortisol [41, 42]. In this study, there was no difference in cortisol levels between the lavender and vehicle groups, but there were differences in terms of depressed mood and blood pressure. Future studies should include participants who are experimentally stressed prior to lavender exposure, or the experimental condition should involve long-term rather than short-term exposure.

## 6. Conclusion

The present study shows that inhalation of lavender oil was effective in decreasing depressed mood in human subjects. Moreover, lavender oil activated OXT neurons in the PVN of the hypothalamus, which contributed to its antidepressant effects. These results suggest that aromatherapy using lavender essential oils might improve depressive mood in healthy individuals.

## Figures and Tables

**Table 1:** Basic statistics and two-way ANOVA (group × time) results.

		Time		F-number		
		Before	After	Group	Time	Interaction
Systolic blood pressure	DW	114.57 (4.63)	110.57 (4.06)	1.46	39.87^∗∗^	15.41^∗∗^
	Lavender	114.29 (3.74)	97.14 (4.27)			
Diastolic blood pressure	DW	67.67 (4.69)	70.86 (5.27)	0.37	2.21	5.83^∗^
	Lavender	69.43 (3.82)	63.43 (3.99)			
Pulse rate	DW	73.71 (3.58)	70.86 (2.72)	0.00	8.52^∗^	2.63
	Lavender	77.14 (3.67)	67.14 (2.30)			
SDS	DW	32.71 (2.19)	33.71 (2.57)	0.05	3.03	7.85^∗^
	Lavender	34.57 (2.75)	30.29 (2.54)			
STAI-S	DW	39.00 (2.65)	36.57 (2.85)	0.50	13.9^∗^	3.16
	Lavender	38.57 (3.55)	31.71 (1.87)			
VAS-hunger	DW	5.39 (1.21)	6.03 (1.09)	0.21	2.70	0.00
	Lavender	6.07 (1.02)	6.69 (1.00)			
VAS-thirst	DW	4.04 (0.80)	5.21 (0.79)	0.07	3.41^†^	0.74
	Lavender	4.74 (1.09)	5.17 (1.05)			
VAS-appetite	DW	6.83 (0.93)	6.86 (0.99)	0.19	3.39^†^	3.09
	Lavender	5.66 (1.07)	6.89 (0.92)			
Cortisol	DW	15.51 (1.12)	13.53 (1.54)	0.15	10.57^∗∗^	0.02
	Lavender	14.94 (0.96)	12.77 (1.46)			

^†^ *p* < 0.10, ^*∗*^*p* < 0.05, ^*∗∗*^*p* < 0.01. DW = distilled water; SDS = Self-Rating Depression Scale; STAI = State-Trait Anxiety Inventory; VAS = Visual Analogue Scale.

**Table 2:** The proportion of lavender-reactive single neurons in oxytocin (OXT) positive and negative single neurons in the paraventricular nucleus.

Number of single neuron cells			
Lavender-reactive (proportion, %)	Lavender-nonreactive	Total
OXT-positive	10 (14.5)^∗^	59	69
OXT-negative	3 (3.7)	78	81
Total	13	137	150

^*∗*^ *p* < .05 versus OXT-negative.

## Data Availability

The data used to support the findings of this study are available from the corresponding author upon request.

## Abbreviations

PVN: Paraventricular nucleus
HPA: Hypothalamic-pituitary-adrenal
CRF: Corticotropin-releasing factor
OXT: Oxytocin
AVP: Arginine vasopressin
BDNF: Brain-derived neurotrophic factor
BSA: Bovine serum albumin
SDS: Self-Rating Depression Scale
STAI: State-Trait Anxiety Inventory
VAS: Visual Analogue Scale
HKRB: HEPES-buffered Krebs-Ringer bicarbonate buffer solution
AM: Acetoxymethyl
ANOVA: Analysis of variance
SEM: Standard errors of the mean
BMI: Body mass index.
